# Neonatal Shock: Current Dilemmas and Future Research Avenues

**DOI:** 10.3390/children12020128

**Published:** 2025-01-24

**Authors:** Vijay Kumar Krishnegowda, Arun Prasath, Viraraghavan Vadakkencherry Ramaswamy, Daniele Trevisanuto

**Affiliations:** 1 Department of Neonatology, Institute of Medical Sciences and SUM Hospital, Bhubaneswar 751003, India; vijaybmc03@gmail.com; 2 Division of Neonatal-Perinatal Medicine, Department of Pediatrics, University of Texas Southwestern Medical Center, Dallas, TX 75390, USA; Arun.Prasath@utsouthwestern.edu; 3 Ankura Hospital for Women and Children, Hyderabad 500072, India; dr.viraraghavan@ankurahospital.com; 4 Department of Woman’s and Child’s Health, University of Padua, 35122 Padua, Italy

**Keywords:** neonate, shock, hypotension, inotrope, vasopressor

## Abstract

Neonatal shock presents a complex clinical challenge and is one of the leading causes of mortality. Traditionally, neonatal shock is equated to hypotension, and therapeutics are often initiated based on low blood pressure (BP) values alone. This fails to address the underlying goal of optimizing the tissue perfusion resulting in both over- and under-treatment of neonatal shock. Also, what defines a normal BP in neonates is still a contentious topic. Further, the most appropriate way of measuring BP in neonates with shock is still debated. Shock secondary to transient circulatory instability and patent ductus arteriosus, conditions that are unique to preterm neonates, have not been researched adequately. Treatment of myocardial dysfunction secondary to perinatal asphyxia, a leading cause of neonatal mortality, is still a conundrum. Quite similarly, there are only a handful of controlled trials evaluating therapeutics in some of the other commonly encountered conditions, namely, septic shock and hypoperfusion secondary to pulmonary hypertension. Even the universally practiced intervention of volume expansion with crystalloid boluses in shock is not backed by high-certainty evidence in neonates. Though the diagnostic modalities of functional echocardiography and near-infrared spectroscopy have aided greatly in the management of neonatal shock in recent years, these have not been proven to be associated with improved critical clinical outcomes such as mortality and major brain injury. To conclude, neonatologists often rely on limited evidence, mostly anecdotal, when treating neonatal shock. This review critically examines the current evidence with respect to various aspects of neonatal shock with an objective to identify the lacunae in the literature that may fuel future research, eventually paving the way to efficacious, safe and evidence-based clinical practice.

## 1. Introduction

Neonatal shock is a critical condition commonly seen in preterm and sick term neonates [1]. It is one of the leading causes of neonatal mortality and short- and long-term morbidities [2]. The underlying etiopathogenesis of neonatal shock is diverse, ranging from delayed transition to extra-uterine life to more severe causes such as sepsis, patent ductus arteriosus (PDA), persistent pulmonary hypertension of the newborn (PPHN), and cardiac dysfunction [3]. These conditions often overlap, complicating both the diagnosis and management of neonatal shock.

Early and accurate identification of the underlying cause of shock is crucial to initiate timely and appropriate interventions. However, diagnosing neonatal shock poses considerable challenges. Conventional clinical parameters often lack predictive accuracy, and the evidence supporting newer diagnostic tools is limited [4]. Furthermore, due to lack of clinical practice guidelines from the major international neonatal academic societies across the globe, treatment practices are predominantly based on anecdotal evidence or expert consensus.

## 2. The Validity of Clinical and Laboratory Parameters for Diagnosis and Management of Neonatal Shock

Identifying neonatal shock often requires the use of multiple tools to assess its complex pathophysiology. Clinicians often rely on both clinical and laboratory parameters to diagnose and treat neonatal shock. The most utilized clinical signs include tachycardia, prolonged capillary refill time (CRT), poor peripheral pulses, low blood pressure (BP) values, difference in core–peripheral temperature (CPT), and urine output [5]. The laboratory parameters that are relied upon include lactate levels and base deficit [6]. Additionally, recent advancements to diagnose and guide therapy include functional echocardiography (TnECHO) and near-infrared spectroscopy (NIRS) [7,8].

### 2.1. Capillary Refilling Time

CRT is a commonly used parameter to assess tissue perfusion. However, CRT is influenced by several factors, and its utility in newborns is less proven. In a systematic review assessing the validity of CRT measurements in various body locations in healthy-term neonates, it was found that CRT varied significantly based on the location as well as the pressing time [9]. It was also observed that there was marked inter-observer variability [10]. The range of normal CRT values in healthy neonates was reported to be from 2.5 s to over 7 s, with the largest variations seen at the foot, though values exceeding 5 s were also noted at the finger, hand, and chest. Additionally, CRT was influenced by ambient, skin, and core temperatures, respectively [9]. In a recent study of healthy-term neonates, where objective variation was minimized using video-editing software, the mean and upper confidence interval (CI) values for CRT were 1.53 and 1.83 s, respectively. The authors concluded that an upper limit of 2 s in term neonates might be a reasonable cut-off indicating poor perfusion [11]. Osborn et al., in their prospective study which included preterm neonates, reported that a CRT of more than 3 s showed limited association with superior vena cava (SVC) flow, with the sensitivity and specificity being 55% (95% CI: 50%, 60%) and 81% (95% CI: 76%, 84%), respectively, in detecting low SVC flow (defined as less than 41 mL/kg/min). However, the study also reported that using a mean arterial blood pressure (MAP) threshold of 30 mm Hg with a central CRT of more than 3 s improved the sensitivity to 78% [12]. Miletin et al., in their observational study, reported that there was no statistically significant difference in the CRT measured on the sternum between those neonates with low or a normal SVC flow. The median (Interquartile Range (IQR)) CRT in neonates with low SVC flow was 2.9 s (2.1–4.2) in this study [13]. Quite similarly, Wodey et al. evaluated CRT in admitted neonates and demonstrated a poor correlation between CRT and echocardiographic findings in neonates without a PDA [14]. To conclude, CRT in isolation may not be a sensitive sign to diagnose hypoperfusion, and its utility may be enhanced by combining with other clinical signs that have been used to diagnose poor tissue perfusion.

### 2.2. Urine Output Assessment

Renal hypoperfusion leads to decreased urine output; hence, it has been widely used as a marker for identifying shock. Oliguria is a marker of acute kidney injury, and it has been shown to be associated with mortality in neonates [15]. However, in preterm neonates, it is confounded by a constellation of factors related to preterm transitional physiology characterized by an initial phase of oliguria followed by a diuretic phase in the early postnatal days [16]. Additionally, due to renal tubular immaturity, preterm neonates may not be sufficiently able to concentrate urine, and hence urine output may not accurately reflect renal perfusion. Moreover, assessing urine output by measuring diaper weight may be inaccurate, and invasive catheters for urine output measurement are not risk- averse as they predispose the neonate to hospital-acquired infections. Miletin et al., in their observational study, concluded that in neonates with low SVC flow (<40 mL/kg/min), there was no significant decrease in the urine output, with a median (IQR) urine output of 2.6 mL/kg/h (0.5–4.7) [13]. Junior et al., in their retrospective cohort study enrolling very low birth weight (VLBW) neonates, concluded that urine output during the transitional period (the first 72 h of life) was a poor predictor for the need for vasopressors, and also of the critical outcome of mortality within 72 h, with the area under the curve (AUC) being less than 0.80 [17]. Contrary to the aforementioned studies, Washio et al., in their prospective observational study evaluating VLBW neonates with late circulatory collapse, reported that the urine output correlated with hemodynamic changes as measured by echocardiography [18]. It is to be noted that the neonates enrolled in this study were more than 7 days of age. The correlation between urine output in the initial days of postnatal life, potential effect modifiers such as administration of intravenous fluid or total parenteral nutrition, and tissue perfusion needs further research.

### 2.3. Heart Rate

Isolated tachycardia is often adjudged an early sign of poor perfusion as it is considered a compensatory mechanism to increase cardiac output to restore tissue perfusion. However, in neonates, heart rate (HR) is influenced by several factors such as temperature, sepsis, medications, and pain, making it a less reliable marker of perfusion [3].

### 2.4. Temperature Difference

CPT difference as a marker of tissue perfusion has several limitations. Lambert et al., in their observational study, noted that there was no statistically significant association between hypotensive neonates whose BP was stabilized with albumin infusion and the CPT difference. Further, the authors reported that a CPT difference of more than 3.4 degrees Celsius was seen in only three of 54 (5.5%) episodes of hypotension in preterm neonates born at a gestational age (GA) of less than 31 weeks [19]. Osborn et al., in their study enrolling preterm neonates of less than 30 weeks GA, reported that a CPT of more than 2 degree Celsius is a poor predictor of tissue perfusion with a sensitivity and specificity of 40% (95% CI: 32%, 48%) and 69% (95% CI: 61%, 77%) for the prediction of low SVC flow, a surrogate marker of cerebral perfusion [12,20]. It should be noted that CPT depends on many factors, including the GA, birth weight, and postnatal age. With such complexity, CPT may not be a good parameter for diagnosing and treating shock in neonates.

### 2.5. Serum Lactate Assessment

Lactate is a byproduct of anaerobic metabolism and is strongly associated with tissue hypoxia. The liver is the primary organ that metabolizes lactate produced in regional tissues and plays a major role in regulating lactate levels. Thus, in cases of liver injury or conditions such as sepsis and anemia, lactate metabolism may be impaired. Lactate as a marker of shock has been widely adopted in several neonatal intensive care units (NICUs) [21]. Nadeem et al., in their retrospective study, concluded that a single lactate level above 5.7 mmol/L obtained on the first postnatal day had a sensitivity and specificity of 100% and 85%, respectively, to predict death or major brain injury (MBI) in preterm neonates born at less than 32 weeks GA [22]. In another study that included all VLBW neonates, the median (IQR) first lactate and highest lactate levels among survivors and non-survivors were reported to be 3.92 (0.67–17.90), 9.38 (2.20–21.10), 3.86 (1.05–20.70), and 10.7 (3.37–29.40) mmol/L, respectively. The accuracy of the first lactate and highest lactate to predict survival was 0.79 (95% CI: 0.17, 0.87) and 0.86 (95% CI: 0.80, 0.91), respectively. The authors concluded that the highest lactate on the first postnatal day had an acceptable accuracy to predict survival [23]. Similarly, Hussain at al., in their retrospective cohort study, enrolled preterm neonates of less than 28 weeks GA with an aim to determine if high lactate levels within the first 12 h of postnatal life as an isolated parameter or when used in combination with Clinical Risk Index for Babies (CRIB) II score could predict mortality. The authors concluded that high levels of lactate in isolation or when used in combination with CRIB II did not improve the predictive ability of CRIB II [24].

Junior et al. reported that lactate measurement in VLBW neonates within the first 72 h of postnatal life did not predict the need for hemodynamic support with an accuracy of 0.67 (95% CI: 0.59, 0.76), nor did it predict death within 3 days (0.66, 95% CI: 0.54, 0.77) or within 10 days (0.70, 95% CI: 0.58, 0.82) [17]. Miletin et al. demonstrated that in VLBW neonates on the first day of postnatal life, lactate levels correlated with low SVC flow (<40 mL/kg/min), with a median (range) lactate level of 3.5 mmol/L (2.8–8.5) in the low SVC flow group when compared to the normal SVC flow group (2.7 mmol/L (1.2–6.9) [13]. In a retrospective study, Wang et al. evaluated the predictive utility of lactate levels within an hour of admission to the NICU and lactate clearance 6 h after fluid resuscitation in neonates diagnosed with septic shock. The authors reported that only when used in combination, a lactate value of >4 mmol/L within an hour of admission and a lactate clearance of >10% after 6 h of volume expansion had an optimal predictive value in prognosticating neonates with septic shock [25]. Sharma et al., in their prospective cohort study, concluded that a lactate level of ≥4.65 correlated with invasive BP measurements with good sensitivity and negative predictive value in late-preterm and term neonates with shock in the first 72 h after shock was diagnosed [26].

To conclude, isolated lactate measurement in hemodynamically stable preterm neonates may not be used as a prognostic parameter. However, in term or preterm neonates diagnosed with shock, serial lactate measurements and integrating lactate levels with other parameters such as CRIB II score may correlate with the disease severity. It is to be noted that lactate may not be an accurate parameter even if used serially in neonates treated with inotropes such as epinephrine as serum lactate levels increase with epinephrine despite normalization of BP and improvement in cerebral blood flow as measured by NIRS [27,28].

### 2.6. Blood Pressure

BP serves as a crucial driver for cardiovascular interventions in preterm neonates, especially when the BP level falls below a pre-defined threshold. Various thresholds have been used to define hypotension in neonates. The commonly used ones are centile-based, a MAP less than the GA and a MAP less than 30 mm Hg. These approaches often undermine the complex physiological and pathological variations of BP trends with advancing postnatal age, especially in preterm neonates [29]. Henceforth, the key questions related to BP in preterm neonates remain unresolved, such as identifying the optimal BP threshold below which perfusion to vital organs is impaired, resulting in short- and long-term morbidities. Moreover, the clinical significance of isolated hypotension (without other signs of tissue hypoperfusion) is yet to be deciphered. Most clinicians treat isolated hypotension, which is often seen in extreme and very preterm neonates in the initial days of postnatal life, and whether interventions targeting isolated hypotension improve outcomes remains an area of active investigation. As of present, there are only three RCTs on this PICO [30,31,32], of which one trial was prematurely stopped [30].

#### 2.6.1. Normative BP Centiles

Studies have published normative BP data based on GA, birth weight, and postnatal age. These studies have marked heterogeneity in terms of population, setting, timing and method of estimation of BP. Elsayed et al., in their study enrolling 206 stable preterm neonates of less than 29 weeks GA, produced a BP nomogram with centiles as measured by intra-arterial BP. They found that the BP centiles increased in the first 72 h of postnatal life, and the trend in increase depended upon the GA [33]. Similarly, Kiss et al. in their retrospective cohort study of stable preterm and term neonates, included 629 neonates and analyzed 134,938 BP values measured by the non-invasive oscillometric method. The study demonstrated that BP in these neonates varies with GA and birth weight [34]. The International Neonatal Consortium performed a systematic review with an aim to develop population-based normative BP data. A total of 30 studies were included in the review with 18 studies including term neonates (n = 14,557) and eight studies enrolling preterm neonates (n = 1334). However, a meta-analysis was not performed due to marked clinical heterogeneity. Though most of the included studies were underpowered, some of them reported a significant correlation between birth weight, GA, and BP [35]. The Consortium emphasized the need to develop universally accepted sub-groups of neonates based on their GA and birth weight, as well as standard postnatal time points. Future studies should include a collaborative multicentric approach and include these parameters with an appropriate statistical plan for analyzing the data [36].

To conclude, BP has marked variability, poor reliability, and limited correlation with clinical outcomes, and with the absence of established evidence-based standards, it is essential to adopt a cautious and least harmful approach. Collaborative efforts by international bodies are needed to conduct robust studies, establish standardized assessment tools, and perform prospective studies to evaluate the utility of BP in the initial days of life for guiding interventions, and their impact on clinical outcomes.

#### 2.6.2. Oscillometric BP Measurement

Indwelling arterial catheters provide accurate measurement of BP and are considered the gold standard for measuring BP. Single-center experiences of using peripheral arterial catheters in extremely low gestational age neonates with promising results have been published [37]. However, most of the units, specifically in low- and middle-income countries, may not have adequate expertise for the same. In addition, indwelling arterial catheters carry the risk of thrombosis, infection, and device failure [38]. Therefore, the usual preferred method of measurement of BP is by non-invasive oscillometric methods. They identify the peak oscillation, estimate the MAP, and by device algorithm derive the systolic and diastolic BP. However, in the neonatal population, the reliability of these devices is affected by many factors. A consensus document was released for standardizing non-invasive BP devices by the Association for the Advancement of Medical Instrumentation, the European Society of Hypertension, and the International Organization for Standardization [39]. The recommendations were as follows: a minimum of 35 participants for BP studies in the neonatal population and a BP difference between the test and approved comparator of <5 mm Hg with a SD of <8 mm Hg [39]. A majority of the neonatal studies conducted so far do not fulfil these requirements. Further, MAP in the neonatal population usually falls within a range of 30–50 mm Hg. Henceforth, the aforementioned recommended standards for difference in BP values may not be practical as this results in a large difference, which would be unacceptable for clinical care in neonates. To address the questions on methods of BP measurement and to formulate recommendations, the International Neonatal Consortium performed a systematic review [40]. For adequate cuff size, four studies were included, and the studies had reliable measurements in comparison to invasive BP values when the cuff width-to-arm circumference ratio size was around 0.5. For the comparison of BP readings between intraarterial and oscillometric methods, eighteen studies were identified. Most of the studies had MAP differences outside the acceptable limits of 5 mm Hg and SD of 5 mm Hg. Only 4 analyses showed MAP measurements within the limits of agreement. These four analyses came from studies with sample sizes of 5 to 14 neonates whose GA and postnatal age varied widely [41,42]. Additionally, a few studies also showed that at low BP readings of less than 30 mm Hg, the reliability of the oscillometric method is poor as it overestimates the MAP [43,44,45]. In addition, the oscillometric method is confounded by movement of the limb where the cuff is applied and the state of infant arousal [41]. These factors hinder the use of non-invasive methods of BP measurement in the management of neonatal shock. Future studies should focus on the various validating methods and innovations with respect to refining the oscillometric BP measurements, especially in the setting neonatal shock.

#### 2.6.3. Role of Diastolic and Systolic BP

Preterm neonates often demonstrate immature myocardial function and shunting of blood through the patent foramen ovale and PDA. This results in increased pulmonary and systemic blood flow and elevated vascular resistance, which places additional strain on the preterm heart, resulting in hypotension. However, relying solely on MAP may not adequately capture the underlying pathophysiological state [46].

For instance, a decrease in systolic BP indicates compromised myocardial function, which can result in reduced stroke volume and cardiac output. In contrast, decreased diastolic BP suggests lower vascular resistance, pointing to a potential low volume state. Although the management strategies for these two scenarios differ considerably, it is not uncommon for both systolic and diastolic BP to be low simultaneously in neonates, complicating the clinical picture. Moreover, just as is the case with MAP, systolic and diastolic BP values are hindered by various other confounding factors such as the method of measuring BP, their reliability (especially with the non-invasive oscillometric method), normative data, GA, and birth weight.

### 2.7. Other Modalities to Diagnose Shock and Guide Therapy: Gaps in Knowledge

#### 2.7.1. Near Infrared Spectroscopy

To address the impact of BP on oxygenation at the tissue level, researchers have utilized NIRS to explore the relationship between BP and tissue oxygenation. NIRS allows the assessment of cerebrovascular reactivity, which is the ability of vasculature to regulate blood flow to meet tissue demands [47]. By using NIRS, researchers have tried to understand the complex interaction between cerebral oxygenation and BP.

NIRS studies have shown that cerebral autoregulation may be intact in stable preterm neonates [48,49]. However, this regulation may be lost in scenarios such as during the immediate period of transitioning from intrauterine to extrauterine life [49], respiratory distress syndrome [50], need for respiratory support [48], and inflammation/infection [51]. Other factors influencing cerebral autoregulation include GA [52] and birth weight [53]. In addition, it has been shown that alterations in the autoregulation as measured by BP values in conjunction with NIRS readings may be associated with intraventricular hemorrhage (IVH) [52,54,55] and poor neurodevelopment outcomes [56].

Binder-Heschl et al. monitored 24-h regional cerebral tissue oxygen saturation (crSO2) in both normotensive and hypotensive preterm neonates. The mean (SD) crSO2 was 76.2% (1.9) for normotensive neonates and 77% (2.2) for hypotensive neonates, respectively. Additionally, the mean (SD) 24-h regional cerebral fractional tissue oxygen extraction was 0.20 (0.31) for normotensive neonates and 0.20 (0.02) for hypotensive neonates, respectively, with no statistically significant difference between the two groups [57]. Similarly, interventions like fluid bolus and dopamine did not result in a change in cerebral oxygenation and extraction [58,59]. However, Erikson et al. demonstrated that dopamine impacts cerebral autoregulation as measured by cerebral oximetry index. The authors reported that the mean (SD) cerebral oximetry index was higher in the dopamine group (0.41 (0.25)) in comparison to the group not treated (0.08 (0.25)) [60]. Da Costa et al., in their prospective observational study on extreme preterm neonates, found that the mean (SD) MAP at which cerebrovascular reactivity is maximized within the first 24 h after birth was 31.3 (4.7) mm Hg, with this value showing an increasing trend with the GA. They also observed that neonates with IVH and mortality had MAP below this value [61]. Pichler et al., in their RCT, studied the utility of NIRS in managing preterm neonates by implementing targeted interventions like echocardiography, fluid administration, or by treating PDA. They observed a nonsignificant reduction in the burden of hypotension with the use of NIRS (defined as MAP less than the GA) [62]. While most studies on NIRS have evaluated cerebral tissue oxygenation, it is to be noted that peripheral tissue oxygenation would impaired in the initial phase of shock. In their prospective observational study, Hoeller et al. evaluated two parameters, namely, peripheral-muscle-fractional-oxygen-extraction (pFOE) and peripheral-muscle-fractional-tissue-oxygen-extraction (pFTOE) in healthy-term and preterm neonates. The authors reported that the difference in the pFTOE and pFOE values widened with impaired tissue perfusion. Though the calculation of pFOE mandates measurement of peripheral-muscle-mixed-venous-saturation (pSVO_2_) which requires an invasive procedure, it may be more reflective of tissue hypoperfusion when compared to pFOE and could be an adjunctive diagnostic modality to the routinely used clinical signs. Further, pFOE might aid in diagnosing shock earlier, before the perfusion to the vital organs is impaired. The utility of pFOE in the diagnosis and management of shock needs to be evaluated in clinical studies enrolling neonates with shock to ascertain if this approach leads to better clinical outcomes [63].

Though NIRS seems to be a promising tool in the diagnosis and management of shock, this needs to be validated in large clinical trials, with the inclusion of critical outcomes such as mortality and MBI.

#### 2.7.2. Functional Echocardiography

In recent years, functional echocardiography (TnECHO) has been widely adopted in NICUs for the diagnosis and management of neonatal shock [64]. It provides an objective tool for assessing the cardiac function. Various echocardiographic parameters used to evaluate cardiac output and tissue perfusion include left ventricular output, right ventricular output, SVC flow, and descending aorta flow. The assessment of volume status involves evaluating parameters such as left ventricular end diastolic diameter, left atrial (LA) diameter, LA/Ao (aortic root) ratio, and collapsibility of the inferior vena cava (IVC) [65]. However, these parameters are poorly validated against standard tools, particularly those parameters used to assess low volume status in neonates. There have been efforts to standardize the use of TnECHO and interpret the findings in neonates [66,67].

Saini et al. reported a higher IVC collapsibility index (IVC CI) in preterm neonates with septic shock compared to controls (53% vs. 20%), although most of the other preload markers did not show significant changes following volume resuscitation [68]. Another study found significantly lower values of TnECHO parameters, including IVC CI, fractional shortening, ejection fraction, cardiac output, and SVC flow in term neonates with cardiogenic or septic shock. Among these, SVC flow correlated significantly with CRT and systolic BP [69]. In a recent study, neonates with septic shock demonstrated a higher IVC distensibility index and shorter isovolumetric relaxation time (IVRT), both of which improved with treatment [70].

The ACCM promotes the use of TnECHO in the management of shock [6]. However, in adults with septic shock, early goal-directed therapy failed to demonstrate improved outcomes and was associated with higher hospitalization costs [71]. Similarly, Raschetti et al. evaluated the impact of TnECHO use on short-term outcomes in extremely preterm neonates. In their study, 229 neonates in the TnECHO group were matched with 229 in the non TnECHO group. There were no significant differences in survival [178/229 (77.8%) vs. 179/229 (78.1%)] or survival without severe morbidity [123/229 (53.5%) vs. 118/229 (51.4%)] between the two groups. However, the TnECHO group received more inotropic medications [77/144 (53.7%) vs. 37/99 (37.8%)]. Thus, the routine use of TnECHO may not improve outcomes but could result in increased use of antihypotensive medications [64]. These findings highlight the knowledge gap in interpreting TnECHO findings and emphasize the need to optimize its use. Future studies are warranted with respect to establishing normative data related to various parameters of TnECHO in neonates.

## 3. Treating Neonatal Shock: The Dilemmas

### 3.1. Should We Treat Isolated Hypotension in the Initial Days?

The normal range of BP in neonates remains ambiguous, resulting in diverse clinical practices, with some clinicians opting to treat isolated hypotension, while others do not. Initial observational studies have indicated a potential association between hypotension or hypotension treatment and adverse outcomes like MBI [72,73]. However, whether these negative effects are a result of the necessity for antihypotensive treatment or of the interventions to increase BP is not conclusive, calling for well-designed RCTs.

Preterm neonates in the first 72 h of life who had hypotension, defined as a MAP less than their GA, also showed clinical signs of poor perfusion in 53% (26/49) of cases. In contrast, among the neonates who displayed clinical features of poor perfusion, only 14% (23/106) had hypotension [74]. Batton et al. conducted a pilot RCT involving extreme preterm neonates born at a GA of 23–27 weeks with isolated hypotension [31]. Of the 58 eligible neonates, only 10 were included in the trial due to slow enrollment, parental unavailability for consent, and lack of physician equipoise. Of the 10 neonates, four were randomized to the permissive arm and six to the active management. None in the permissive group and two out of the six neonates in the active group died and had MBI. Another RCT randomized neonates into three groups: the active arm (treatment initiated for isolated hypotension if the MAP is below 30 mm Hg for more than 15 min), the moderate arm (MAP below GA for more than 15 min), and the permissive arm (MAP below 19 mm Hg for more than 15 min or had impaired perfusion based on clinical and biochemical assessments). Across the groups, there were no significant differences in outcomes, including mortality and MBI. However, the study was underpowered [32]. Furthermore, a multicentric RCT was conducted to evaluate whether the restricted use of inotropes for isolated hypotension during the first 72 h impacts survival without MBI in preterm neonates born at a GA of less than 28 weeks. This trial was terminated early due to challenges in recruitment, with the final analysis including 58 neonates. The groups within this study were found to be comparable, and there were no statistically significant differences between the two groups for any of the outcomes [30]. Considering that these small trials did not conclusively yield any major findings, large multi-center trials evaluating this PICO are warranted. These trials had highlighted the various difficulties in conducting RCTs addressing this PICO, and these bottlenecks may be diligently addressed in future trials. EPIPAGE 2, a multicentric cohort study, included preterm neonates less than 29 weeks with MAP less than GA without any clinical or biochemical indicators of hypoperfusion, and evaluated the outcomes in two groups: one receiving no treatment and the other receiving active treatment. Each group included 119 neonates by propensity score matching. The study showed that treated neonates had a higher survival rate without major morbidity (OR 1.67, 95% CI: 1.0, 2.78) and a lower rate of severe cerebral abnormalities. (10.1% vs. 26.5%) [75] (Table 1).

#### Does Hypotension Lead to Adverse Outcomes?

Several observational studies have provided conflicting results regarding the relationship between hypotension and its potential consequences. While some studies report an association between hypotension and an increased risk of IVH [72,73,83,84,85] or periventricular leukomalacia, others have found greater fluctuations in BP due to interventions to treat isolated hypotension to be the causative factor for MBI [72,86,87]. Various other studies suggesting that hypotension is unrelated to white matter injury and PVL have been published in the literature [88,89,90]. Logan et al., in their prospective observational study enrolling 1041 preterm neonates born at a GA of less than 28 weeks, examined the association between BP in the first 24 h of postnatal life and various adverse outcomes. The study did not find a significant association between different indicators of hypotension and white matter injury or cerebral palsy [89].

Active management of isolated hypotension may not consistently correlate with improved outcomes. However, the largest cohort study reported a strong association between not treating isolated hypotension and poor short-term outcomes [80]. In this study, the threshold for intervention was defined as MAP pressure below the GA in weeks minus 5 mm Hg, persisting for at least 15 min within the first 24 h of life. Thus, active intervention under these criteria may be considered. Additionally, a subgroup analysis from two non-randomized studies suggested that intervention for isolated hypotension in very preterm neonates beyond the first 24 h but within the first 72 h of birth was associated with a reduction in the incidence of necrotizing enterocolitis (NEC) and MBI [75,76]. These findings indicate that there might be a subset of very preterm neonates who may benefit from treatment of isolated hypotension between 24–72 h of postnatal life. Further, a safety net for treating isolated hypotension in all very preterm neonates of any gestational or postnatal age may be a MAP of less than the GA minus 5 mmHg. These findings need to be validated in future RCTs.

### 3.2. Does Normal Saline Bolus Improve Outcomes in Extreme Preterm Neonates with Hypotension in the Initial Days of Life?

Multiple reports have shown that 0.9% sodium chloride is the most commonly used crystalloid solution for volume expansion and that treatment of hypotension, poor perfusion, and metabolic acidosis with volume expansion may not result in improved outcomes in extremely and very preterm neonates [91,92]. In a neonates with normal intravascular volume and who are hypotensive from transient circulatory instability (TCI), volume replacement may not improve MAP, though it may transiently improve left ventricular output [93]. A study in preterm neonates reported that early fluid boluses, especially within the first 24 h of life, can result in an increased incidence of IVH without any beneficial effect on the systemic perfusion [94]. Further, fluid boluses may also increase the incidence of PDA and the need for home oxygen [95]. Sehgal et al., in their case series including 16 extreme preterm neonates with hypotension, observed that normal saline boluses were associated with decreased pulmonary compliance, increased requirement of peak inspiratory pressure (PIP), and other adverse pulmonary mechanics. The authors postulated that these adverse effects could be attributed to a pulmonary capillary leak in these vulnerable neonates whose vascular tone is inherently compromised, increased back pressure due to an immature myocardium, and excessive cytokine production initiated by the ventilator-induced lung injury due to high PIP [96]. Ewer et al., in their case-control study, reported that excessive volume administration (≥30 mL/kg) in the first 48 h of life in preterm neonates born at a GA of 27–28 weeks may be associated with an increased risk of mortality [97]. A Cochrane review also reported no significant benefit of routine volume expansion in preterm neonates with early cardiovascular compromise [93].

#### What Are Management Strategies for Transitional Circulatory Instability?

Even though there are no universal criteria for diagnosing probable TCI in preterm neonates, parameters like BP, clinical tissue perfusion markers (e.g., capillary refill time, urine output), biochemical markers like lactate, and TnECHO measurements (e.g., cardiac flows and IVC) have been used to identify probable TCI. Although the decision to treat or not treat probable TCI remains a matter of debate, most clinicians continue to treat it. However, treatment approaches for TCI vary widely. To evaluate the role of treatment in TCI, we performed a systematic review and a network meta-analysis of 14 RCTs involving mostly neonates born before 29 weeks of gestation [98]. In most studies, low BP was the primary criterion for initiating treatment. The network meta-analysis indicated that epinephrine possibly decreased the risk of MBI when compared to dobutamine and milrinone, and possibly decreased the likelihood of NEC when compared with dopamine, dobutamine, hydrocortisone, and milrinone. The results also indicated that dopamine was possibly associated with a lesser risk of NEC when compared with dobutamine [98]. However, the evidence certainty was very low for all the comparisons. Given the uncertainty in the diagnostic criteria for TCI, there is an urgent need for objectively defining TCI.

### 3.3. How to Manage Cardiogenic Shock Secondary to Perinatal Asphyxia?

Neonatal encephalopathy resulting from perinatal asphyxia is associated with high mortality and morbidity [99]. It is commonly associated with multiorgan dysfunction, and the severity of organ involvement is proportional to the extent of the hypoxic insult. In clinical trials, cardiovascular involvement, as indicated by the need for inotropes, ranges from 33–77% in those treated with therapeutic hypothermia and 25–83% in those who are managed conservatively [100]. However, the relationship between the extent of cardiac involvement and neurodevelopmental outcomes remains unclear.

Perinatal asphyxia affects the myocardium by primary hypoxic insult, and the myocardial injury may be accentuated further due to the redistribution of blood flow to the brain as a compensatory mechanism. Asphyxia may also be associated with meconium aspiration syndrome, leading to PPHN. This can lead to right sided heart failure and decreased blood flow to the lungs, further lowering left ventricular preload and eventually resulting in systemic hypotension. Moreover, therapeutic hypothermia by itself may worsen pulmonary vasoconstriction, increase pulmonary vascular resistance, and compromise right ventricular function further. At the same time, systemic vasoconstriction raises systemic vascular resistance, which could also negatively impact the left ventricular function.

Crystalloid boluses are used widely by clinicians in neonates with asphyxia induced myocardial dysfunction without any robust evidence for the same. However, trials from preclinical, pediatric, and adult critical care have allowed us to reconsider these practices in neonates. Preclinical studies investigating the effects of volume expansion in piglets following asphyxia does not support its use unless there was evidence of volume loss [101,102]. At the same time, it was found to increase the incidence of pulmonary edema [101]. No clinical trials have examined the role of bolus administration in this population. One retrospective study evaluating neonates born at more than 34 weeks gestation and who required resuscitation in the delivery room indicated that crystalloid bolus did not improve the arterial pH, pCO_2_, heart rate, or mortality. However, those neonates who received bolus had lower arterial cord pH and high base deficits, with a prolonged period of chest compression and adrenaline administration [103]. Thus, receiving a bolus may indicate the severity of illness and may not provide an objective immediate benefit. A multi-center pragmatic trial evaluated the prevalence and practice of fluid bolus administration in neonates. A total of 163 neonates with probable hemodynamic instability received a fluid bolus. The most frequently administered fluid was 0.9% sodium chloride (129/163; 79%), with 71% (116/163) of boluses given at a dose of 10 mL/kg and a median administration time of 30 min. The prevalence of fluid therapy ranged across centers from 0% to 28.6% of admitted neonates. The most common indications for bolus administration were hypotension (56/163; 34%), poor perfusion (20/163; 12%), and metabolic acidosis (20/163; 12%). In 66 out of the 163 neonates (40%) who received volume expansion, there was minimal or no clinical improvement [91].

Giesinger et al. analyzed the right ventricular function by echocardiography in 53 neonates with perinatal asphyxia. The study found right ventricular systolic performance was lower in these neonates. Additionally, the tricuspid annular plane systolic excursion and right ventricular fractional area change was associated with adverse outcomes. The study also found that neonates who had adverse outcomes had an increased need for inotropes at 24 h (12/18; 67%) in comparison to those without any adverse outcomes (10/35; 29%) [104]. Similarly, Al Balushi et al., in their retrospective cohort study, reported that hypotension was frequently observed in neonates with perinatal asphyxia, and that hypotension was associated with MBI [105].

Dopamine, administered at a dose of 10 mcg/kg/min and escalated to 20 mcg/kg/min if the SVC flow falls below 40 mL/kg/min, did not increase the SVC flow. However, dopamine does lead to a significant increase in BP in preterm neonates [106]. In neonates with perinatal asphyxia, higher SVC flows during hypothermia are associated to poor short- and long-term outcomes, likely due to disruptions in brain circulation in those with severe injury [107,108]. Therefore, it may be preferable to use inotropes that improve systemic BP without altering the SVC flow when treating neonates with perinatal asphyxia.

The RCT by DiSessa et al. showed that low-dose dopamine (2.5 mcg/kg/min) resulted in improved cardiac performance and increased systolic BP in neonates with severe asphyxia [109]. However, another RCT that utilized a low dose of dopamine of 3 mcg/kg/min for 48 h found no additional benefits in terms of mortality or other short-term outcomes [110]. A case-control study evaluated the outcomes of neonates with neonatal encephalopathy who were treated with therapeutic hypothermia and who received dopamine infusion following volume resuscitation, comparing them to those who did not receive dopamine. After 9 h of treatment, the MAP in the dopamine group increased by 9 mm Hg, which was comparable to the control group, and the BP sustained thereafter. However, no associations were found between dopamine use and other outcomes including death or MBI [111].

In an echocardiography study which assessed the cardiac function in transient myocardial dysfunction, it was reported that the use of dopamine at a dose of 4 to 10 mcg/kg/min in six neonates with myocardial dysfunction and hypotension resulted in an increase in BP, improved the cardiac output and stabilized the heart rate. The MAP continued to increase with the use of dopamine and the BP values were sustained after the discontinuation of dopamine [112].

In a retrospective study which enrolled 64 neonates with neonatal encephalopathy who underwent therapeutic hypothermia, the electrocardiographic and echocardiographic findings in relation to long-term outcomes were assessed. During the study period, the unit protocol used dobutamine as the first-line drug, followed by dopamine and epinephrine. The findings indicated that neonates who received lower doses and shorter durations of dopamine and dobutamine had a reduced risk of death or moderate to severe neurodevelopmental disabilities. Furthermore, exposure to epinephrine was linked to poorer outcomes [108].

Relative adrenal insufficiency is defined as relative low levels of cortisol secretion for the severity of illness. To assess the relative adrenal insufficiency, Kovacs et al. included neonates with hemodynamic instability undergoing therapeutic hypothermia and assessed their cortisol levels. The authors reported that 22.8% of these neonates had low cortisol levels [113]. Kovacs et al. followed this with an RCT performed to assess the effect of the addition of hydrocortisone with dopamine in neonates with volume resistant shock. It was seen that the addition of hydrocortisone was effective in raising BP and assisted in weaning dopamine earlier. Neonates in the dopamine-alone group had lower electrographic seizures when compared to those who received dopamine along with hydrocortisone [114]. From the existing literature, it is evident that there exists significant uncertainty as to how to treat hemodynamic compromise in asphyxiated neonates with shock. Further RCTs are encouraged to evaluate other inotropes in the setting of myocardial dysfunction and hypoperfusion secondary to perinatal asphyxia.

### 3.4. Inotrope of Choice in Patent Ductus Arteriosus and PPHN with Shock

PDA allows blood to shunt from systemic circulation to pulmonary circulation and can lead to diastolic hypotension. A hemodynamically significant PDA can manifest with a myriad of clinical signs and symptoms including increasing respiratory support requirement, congestive heart failure, apnea, necrotizing enterocolitis, and poor weight gain [115]. In those preterm neonates with hemodynamically significant PDA causing clinical manifestations, pharmacological treatment with either ibuprofen or paracetamol is the first-line therapy [116]. In those with systemic hypotension, inotropes may be warranted. If the PDA does respond to pharmacological interventions, surgical ligation is the standard of care, especially if the symptomatology is severe and the neonate requires invasive mechanical ventilation [117]. Post-ligation syndrome (PLS) is a common occurrence in the postoperative period after PDA ligation. Though the pathophysiology of PLS has been understood to be multifactorial, a sudden increase in left ventricular afterload after PDA ligation in conjunction with relative adrenal insufficiency has been postulated to be the driving factor. Though low-dose prophylactic milrinone initiated in the immediate postoperative period in those with a left ventricular output of less than 200 mL/kg/min has been the traditional practice, the evidence certainty for this strategy is uncertain. Routine administration of hydrocortisone perioperatively has also been suggested [118].

Bouissou et al., in their case series, including 17 preterm neonates of GAs less than 32 weeks with PDA associated hypotension, evaluated the effect of dopamine infusion on tissue perfusion (measured by SVC flow). The authors reported that after 2 h of initiation of dopamine infusion, the MAP, the pulmonary arterial pressure, and the SVC flow increased significantly from baseline. The authors postulated that dopamine decreases the left-to-right shunting across the PDA due to a relative increase in pulmonary vascular resistance when compared to systemic vascular resistance [119]. These findings were supported by Liet et al. in their observational study in preterm neonates with PDA and shock. Quite similar to the study by Bouissou et al., there was an increase in MAP by 41% and the mean pulmonary artery pressure by 43%. The authors concluded that dopamine has variable effects on systemic-pulmonary MAP ratio with half of the studied neonates showing an increase in pulmonary arterial pressure relative to the systemic MAP [120]. When managing neonates with PPHN and systemic hypoperfusion, therapeutics are initiated to lower the pulmonary arterial pressure and decrease the right-to-left shunting across the PDA. Despite using pulmonary vasodilators such as inhaled nitric oxide and others, many of the neonates do not respond with ensuing systemic hypotension [121]. Vasopressin and noradrenaline have shown promising results in treating hypotension secondary to PPHN [122,123]. This has been postulated to be due to their differential mechanism of action with respect to the receptors in the systemic and pulmonary vasculature. Both of these drugs have been shown to decrease pulmonary pressure in conjunction with increasing systemic blood pressure.

All the aforementioned studies are small-sized cohort or case-series studies, and an adequately powered RCT is warranted to determine which inotrope is the most efficacious in treating hypotensive neonates with PDA and PPHN.

### 3.5. Inotrope of Choice in Septic Shock

The hemodynamic response in neonatal sepsis is highly variable compared to the adult and pediatric populations. Neonates with sepsis may be normotensive due to high systemic vascular resistance and can present with tachycardia and poor perfusion alone. Septic neonates can also present with hypotension with either adequate perfusion (warm shock, vasodilation) or inadequate perfusion (cold shock, vasoconstriction). The diagnosis and management of septic shock is further complicated by coexisting morbidities and other confounders such as PDA, PPHN, respiratory distress syndrome, and mechanical ventilation [124]. In term neonates, aggressive fluid resuscitation is recommended if there are signs of intravascular volume depletion (20–40 mL/kg) [125]. However, there is insufficient evidence for volume expansion in preterm neonates, and rapid volume expansion is also associated with an increased risk of IVH. In cases with no evidence of volume loss, it is not unreasonable to administer a single bolus of 0.9% saline 10 mL/kg over 30–60 min and to consider starting inotropic support if hypotension is refractory [125].

Dopamine is generally used as the first-line agent in many neonatal units [126]. A randomized, blinded controlled trial by Pellicer et al. showed that low-dose epinephrine was as effective as low-to-moderate-dose dopamine in low birth weight neonates. A recent pilot RCT by Garegrat et al. compared norepinephrine to epinephrine, and the authors reported that either of these drugs had a similar effect on the resolution of hypotension, and there were no statistically significant differences in mortality between the two groups [27]. In neonates with depressed myocardial function, dobutamine can be an alternate choice as it aids in improving the SVC blood flow and cardiac output when compared to dopamine [106,127]. Care should be taken while dobutamine is initiated since it is associated with a heightened risk of tachycardia. Tachycardia alone can lead to decreased cardiac output due to underfilling of the left ventricle, which normally happens during the diastolic phase. Currently, no neonatal studies support the use of milrinone in septic shock. Agents like methylene blue, when compared to terlipressin, a synthetic analogue of vasopressin, were shown to improve systemic vascular resistance and improvement in BP without considerable side effects in preterm neonates with refractory septic shock not responding to norepinephrine infusion [128]. In conclusion, even for a commonly encountered scenario such as septic shock, the evidence base for its management is limited in both term and preterm neonates.

### 3.6. Other Commonly Used Adjunctive Therapies in Septic Shock

#### 3.6.1. When to Initiate Hydrocortisone in Neonates with Septic Shock?

Hydrocortisone is generally initiated in preterm neonates refractory to volume expansion and vasopressors. Cortisol levels remain low in preterm neonates until 32 weeks of gestation. There is no consensus on the definition or diagnosis of relative adrenal insufficiency in preterm neonates [129,130]. Corticosteroids can improve BP by improving the myocardial contractility, increasing the vascular tone, decreasing the capillary leak, and increasing the vascular responsiveness to catecholamines. Though hydrocortisone has been shown to improve the hemodynamic profile in neonates with septic shock, it may not improve the critical outcome of survival [131]. Various studied doses of hydrocortisone for neonatal hypotension are described in Table 2.

These doses resulted in improved BP, with the neonates being weaned off vasopressors earlier [132,133,134,135,136]. In refractory hypotension, one suggested approach to hydrocortisone administration is based on the response to the initial dose as well as the GA of the neonate. If an initial hydrocortisone dose of 1 mg/kg does not result in improving the systemic BP in 2–4 h, the institution of further doses of hydrocortisone may be reconsidered [137]. In cases of response, subsequent dosing of 0.5 mg/kg every 6–8 hourly in late preterm and term neonates and 0.5 mg/kg every 12 hourly in those born at less than 34 weeks’ GA is suggested. The differing dosing regimen is due to the fact that late preterm neonates and term neonates metabolize hydrocortisone faster [137]. Hydrocortisone was also effective in raising the MAP and in decreasing the need for inotrope requirement in asphyxiated neonates undergoing therapeutic hypothermia [114]. However, the most important and unanswered aspect related to hydrocortisone use in neonates with shock is its timing of initiation. To date, no RCT has been published that has evaluated the effect of earlier administration of hydrocortisone versus later administration of hydrocortisone in neonates with shock. This PICO needs to be evaluated in a multi-center RCT.

**Table 2:** Different doses of hydrocortisone used in neonatal shock.

Study/Year	Dose
Ng et al. [136] 2016	1 mg/kg Q4h for 5 d of hydrocortisone or 0.5 mg/kg of dexamethasone
Seri et al. [125] 2001	2–6 mg/kg/d for 1–3 days
Ng et al. [134] 2006	1 mg/kg Q8h for 5 days
Noori et al. [135] 2006	Loading 2 mg/kg, maintenance 1 mg/kg Q12h for 2 days

#### 3.6.2. Role of Sodium Bicarbonate

Acidosis results in the newborn’s myocardium being more resistant to inotropes aimed to increase its contractility when compared to that of the adult population [138]. Sodium bicarbonate is generally not recommended for routine use in neonates due to no proven clinical benefits and potential harmful effects that include increased intracellular acidosis, fluctuating cerebral blood flow leading to increased risk of IVH, especially in extremely preterm neonates, and impairment of myocardial function, which could further worsen the hypotension [139,140]. Additionally, neonates at high risk of myocardial dysfunction may be particularly vulnerable to the adverse effects of bicarbonate therapy, as it could worsen myocardial injury. Another concern is the dose, dilution, and rate of administration of bicarbonate infusion, which is more arbitrary than driven by the studies [141]. A Cochrane review concluded that there is insufficient evidence that infusion of sodium bicarbonate reduces the mortality of neonates receiving resuscitation in the delivery room [142]. Another Cochrane review on bicarbonate administration in preterm neonates with metabolic acidosis included two small RCTs and did not find any effect on the outcomes of mortality and IVH. The authors concluded that there is insufficient evidence for the use of sodium bicarbonate in preterm neonates with metabolic acidosis of any etiology [143]. Thus, the focus should remain on treating the etiology contributing to shock and metabolic acidosis.

## 4. Conclusions

Diagnosing neonatal shock remains a significant challenge due to the limited predictive accuracy of commonly used clinical and biochemical parameters. However, a combination of two or more parameters may assist in the right identification of neonates with hypoperfusion who may benefit from therapy. Substantial evidence is still needed to determine whether isolated hypotension in the early neonatal period is clinically significant, and whether its treatment improves clinical outcomes. Emerging diagnostic and therapeutic modalities like NIRS and TnECHO are routinely used in the setting of neonatal shock, but these modalities need to be validated in larger studies. There is a significant knowledge gap with respect to selection of anti-hypotensive therapies based on the underlying pathophysiology in neonates. Commonly used interventions, such as volume expansion and bicarbonate administration, are largely based on expert consensus rather than robust clinical evidence. Thus, adequately powered and well-designed randomized controlled trials are urgently needed to address the numerous unanswered questions surrounding the diagnosis and management of neonatal shock.

## Figures and Tables

**Table 1:** Characteristics of studies which included preterm neonates with permissive hypotension.

Study Year Design	GA/ BW	PNA	n	Method	Definition	Outcome	Mortality	IVH/PVL	NEC	ROP	ND
Batton [31] 2012 RCT	23–27 wks	<24 h	10 T: 4 P: 6	IABP	1–6 h—24 mm Hg 7–12 h—25 mm Hg 13–18 h—26 mm Hg 19–24 h—27 mm Hg	Feasibility study, study was not feasible	No differences T: 0/4 (0%) P: 2/6 (33%)	No differences T: 0/4 (0%) P: 2/6 (33%)	No differences T: 0/4 (0%) P: 0/6 (0%)	NS	NS
Pereira [32] 2017 RCT	<29 wks	<72 h	60 T: 39 P: 21	Both	T: <30 mm Hg, <GA P: signs of poor perfusion or <19 mm Hg	No differences in mortality or MBI	No differences T: 7/39 (18%) P: 2/21 (9.5%)	No differences IVH T: 6/39 (15.3%) P: 1/21 (4.7%) cPVL T: 0/39 (0%) P: 0/21 (0%)	No differences T: 9/39 (23%) P: 6/21 (28%)	No differences T: 3/39 (7.6%) P: 2/21 (9.5%)	NS
Dempsey [30] 2021 RCT	<28 wks	<72 h	58 T: 29 P: 29	IABP	<GA for 15 min	No differences in mortality or MBI	No differences T: 6/29 (21%) P: 7/29 (24%)	No differences IVH: T: 5/29 (17%) P: 2/29 (7%) PVL T: 2/29 (7%) P: 2/29 (7%)	No differences T: 1/29 (3%) P: 4/29 (14%)	NS	NS
Aladangady [76] 2023 Retrospective	23–29 wks	<72 h	671 T: 263 P: 408	Both	<30 mm Hg	Death and IVH was similar; P: NEC was higher	No differences T: 56/263 (21.3%) P: 104/408 (25.5%)	No differences T: 21/263 (8%) P: 51/408 (12.5%)	P: Increased T: 37/263 (14.1%) P: 85/408 (20.8%)	NS	NS
Batton [77] 2013 Prospective	23–27 wks	<24 h	367 T: 203 P: 164	Both	15 definitions of low BP based on GA, <5th percentile and <25 mm Hg	T: Increased mortality, increased IVH grade 3/4 or any IVH, increased ROP	T: Increase T: 66/203 (33%) P: 36/164 (22%)	T: IVH increased T: 44/203 (22%) P: 18/164 (11%) cPVL: no differences T: 11/203 (5%) P: 7/164 (4%)	No differences T: 16/203 (8%) P: 11/164 (7%)	T: Increase T: 31/203 (15%) P: 13/164 (8%)	NS
Batton [78] 2015 Prospective	23–27 wks	<24 h	356 T: 198 P: 158	Both	Need for treatment, BP < GA	T: Increased Death or any NDI at 18–22 months	T: Increased T: 57/198 (28%) P: 27/158 (17%)	T: Increase IVH/PVL T: 49/198 (25%) P: 19/158 (12%)	No differences T: 8/198 (4%) P: 6/158 (3.7%)	NS	No differences T: 30/198 (15.1%) P:15/158 (9.4%)
Batton [29] 2009 Retrospective	23–25 wks	<72 h	101 T: 70 P: 31	Both	≥3 MAP ≤25 mm Hg	neonates with low BP (± treatment) had worse ND	No difference T: 45/70 (64%) P: 23/31 (74%)	No difference T: 16/70 (23%) P: 9/31 (29%)	No difference T: 3/70 (4%) P: 4/31 (13%)	No difference T: 28/70 (40%) P: 13/31 (43%)	No difference T: 48/70 (68%) P: 19/31 (61%)
Durrmeyer [75] 2017 Prospective	<29 wks	<72 h	238 T: 119 P: 119	Both	<GA in weeks	T: Increased survival without severe morbidity T: Low MBI	T decrease T: 20/119 (16.8%) P: 27/119 (22.6%)	T decrease T: 12/119 (10.1%) P: 31/117 (26.5%)	No differences T: 4/119 (3.4%) P: 6/118 (5.1%)	No differences T: 1/99 (1%) P: 2/92 (2.2%)	NS
Dempsey [79] 2009 Retrospective	<1000 gm	<72 h	104 T: 18 P: 34 N: 52	Both	P: BP < GA with good perfusion T: BP < GA but poor perfusion	T: increased [OR 9.7, 95% CI: 2.6–36] T: Increase mortality or MBI, surgical NEC, or GI perforation	T increase T: 13/18 (72%) P: 4/34 (12.5%) N: 10/52 (19%)	T increase IVH T: 5/18 (27.7%) P: 4/34 (12%) N: 2/52 (3.8%)	No differences T: 2/18 (11%) P: 3/34 (9%) N: 4/52 (8%)	No differences	No differences
Faust [80] 2015 Retrospective	<32 wks	<24 h	4907	Both	<median as per GA	Lowest MAP associate with mortality 67/1064 (6.3%), IVH 255/1064 (24%) and BPD 227/1064 (21.3%)	T: No differences [OR 1.48, 95% CI 0.92, 2.38]	T: Increase [OR 1.86, 95% CI: 1.43, 2.42]	NS	No differences	NS
Gogcu [81] 2020 Case-control study	<1000 gm	<24 h	100 T: 34 P: 66	Both	<GA in weeks	Hypotension requiring treatment associated with increased SNHL risk	NS	T: 9/34 (27%) P: 9/66 (14%)	NS	NS	15/25 (60%) Hearing impairment required treatment; 19/75 (25%) no hearing impairment group required treatment
Ahn [82] 2012 Retrospective	<1000 gm	<72 h	261 T: 47 P: 104 N: 110	Both	<GA in weeks	T: Increase in mortality, IVH, BPD	T increase T: 24/47 (51%), P: 17/104 (16%) N: 13/110 (12%)	T increase IVH >3 T: 18/40 (45%) P: 18/104 (17%) N: 9/110 (8%)	T increase T: 6/34 (18%) P: 10/99 (10%) N: 7/109 (6%)	T increase T: 13/33 (39%) P: 26/97 (27%) N: 23/104 (22%)	MDI < 75 P: 3/36 (8%) N: 3/30 (10%) CPP: 15/83 (18%) N: 14/89 (16%)

**Abbreviations:** GA: gestation age, BW: birth weight, PNA: postnatal age, h: hours, min: minutes, IVH: intraventricular hemorrhage, PVL: periventricular leukomalacia, cPVL: cystic periventricular leukomalacia, NEC: necrotizing enterocolitis, MBI: major brain injury, ROP: retinopathy of Prematurity, BPD: bronchopulmonary dysplasia, ND: neurodevelopment, RCT randomized controlled trial, wks: weeks, IABP: intraarterial blood pressure, T: treatment group, P: permissive group, N: normal group, GA: gestational age, NDI: neurodevelopmental impairment, GI: gastrointestinal, OR: Odds ratio, CI: confidence interval outcome, MAP: mean airway pressure, SNHL: sensorineural hearing loss.

## Abbreviations

The following abbreviations are used in this manuscript:

AUC	Area under the curve
BP	Blood pressure
CI	Confidence interval
CPT	Core-peripheral temperature
CRIB	Clinical Risk Index for Babies
CRT	Capillary refill time
GA	Gestational age
IQR	Interquartile range
IVC	Inferior vena cava
IVH	Intraventricular hemorrhage
MBI	Major brain injury
NICU	Neonatal Intensive Care Unit
NIRS	Near-infrared spectroscopy
PDA	Patent ductus arteriosus
MAP	Mean arterial blood pressure
PPHN	Persistent pulmonary hypertension of the newborn
RCT	Randomized controlled trial
SVC	Superior vena cava
TCI	Transient circulatory instability
TnECHO	functional echocardiography
